# Inter- and intramolecular enantioselective carbolithiation reactions

**DOI:** 10.3762/bjoc.9.36

**Published:** 2013-02-13

**Authors:** Asier Gómez-SanJuan, Nuria Sotomayor, Esther Lete

**Affiliations:** 1 Departamento de Química Orgánica II, Facultad de Ciencia y Tecnología, Universidad del País Vasco / Euskal Herriko Unibertsitatea UPV/EHU, Apdo. 644, 48080 Bilbao, Spain

**Keywords:** alkenes, asymmetric synthesis, carbolithiation, carbometallation, enantioselectivity, lithium

## Abstract

In this review we summarize recent developments in inter- and intramolecular enantioselective carbolithiation reactions carried out in the presence of a chiral ligand for lithium, such as (−)-sparteine, to promote facial selection on a C=C bond. This is an attractive approach for the construction of new carbon–carbon bonds in an asymmetric fashion, with the possibility of introducing further functionalization on the molecule by trapping the reactive organolithium intermediates with electrophiles.

## Introduction

The carbolithiation reaction has attracted considerable interest among synthetic organic chemists, as it offers an attractive pathway for the efficient construction of new carbon–carbon bonds by addition of an organolithium reagent to nonactivated alkenes or alkynes, with the possibility of introducing further functionalization on the molecule by trapping the reactive organolithium intermediates with electrophiles. Several reviews have covered the synthetic applications of this kind of reaction [1–8].

When alkenes are used, up to two contiguous stereogenic centers may be generated, which may be controlled by using chiral ligands for lithium, thus opening new opportunities for their application in asymmetric synthesis. The naturally occurring alkaloid (−)-sparteine, which has been until recently inexpensive and commercially available, is the most widely used chiral ligand in enantioselective carbolithiation reactions. These reactions can be carried out either in an inter- or intramolecular fashion, though only a few examples have been described, due to the difficulty of enantiofacial differentiation for a nonactivated alkene. The intramolecular version has found application in the synthesis of both carbocycles and heterocycles, with a high degree of regio- and stereoselectivity in the formation of five-membered rings, although its application to larger rings is still not general.

The present review will survey some recent advances in the application of inter- and intramolecular enantioselective carbolithiation reactions. The review will not attempt to provide exhaustive coverage of the literature, but it is intended to focus on examples in which a stereogenic center is created in the chiral-ligand-mediated carbolithiation reaction of achiral substrates.

## Review

### Intermolecular carbolithiation reactions

**Scheme 1:**
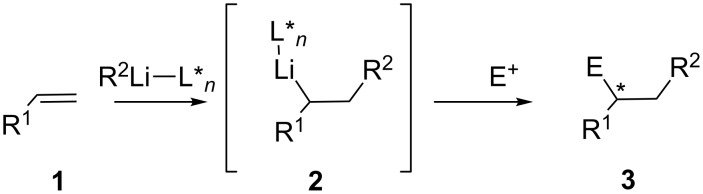
Intermolecular carbolithiation.

Enantioselective versions of intermolecular carbolithiation reactions can be carried out under the influence of a chiral ligand for lithium (L*_n_) to promote facial selection on a carbon–carbon double bond, though only a few examples have been described, due to the difficulty of enantiofacial differentiation for an nonactivated alkene. Besides, a major concern for the synthetic use of intermolecular carbolithiation reactions is the polymerization of the intermediate organolithium **2**. Thus, successful examples have been developed for substrates with substituents on the alkene that may stabilize the resulting organolithium in different ways, to avoid this polymerization (Scheme 1).

**Scheme 2:**
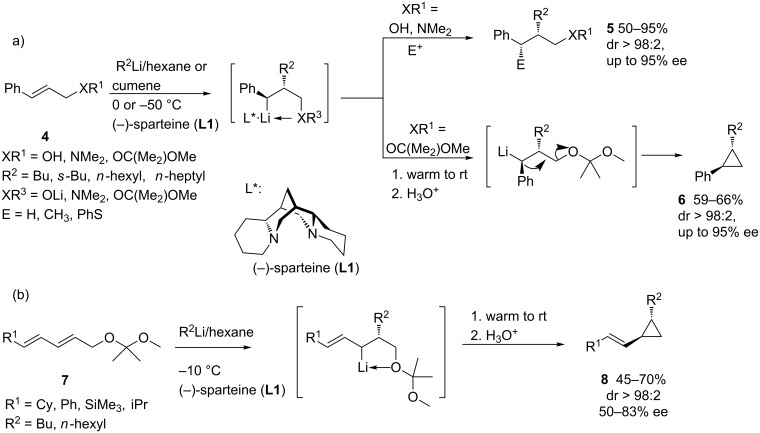
Carbolithiation of cinnamyl and dienyl derivatives.

The first examples of intermolecular enantioselective carbolithiations of styrene derivatives were reported by Normant and Marek [9], taking advantage of the complex-induced proximity effect (CIPE). Thus, the addition of primary and secondary alkyllithiums to (*E*)- and (*Z*)-cinnamyl alcohols and amines **4** in the presence of stoichiometric or substoichiometric amounts of (−)-sparteine (**L1**) led to the corresponding alkylated products **5** in high enantiomeric excess (Scheme 2a). The chiral benzylic organolithium intermediates, which are stabilized by coordination with a Lewis-basic substituent, react with different electrophiles in a highly diastereoselective manner. On the other hand, when acetals derived from cinnamyl alcohols are used as substrates, cyclopropanes **6** can be obtained in high enantiomeric excess by warming the reaction mixture to room temperature, since the resulting benzyllithium intermediates undergo 1,3-elimination [10]. In a similar fashion, the asymmetric carbolithiation of dienyl systems **7** can also be carried out with alkyllithiums in the presence of substoichiometric amounts of (−)-sparteine, thus obtaining *trans*-disubstituted vinylcyclopropanes **8** in moderate to good enantiomeric excesses (Scheme 2b) [11].

**Scheme 3:**
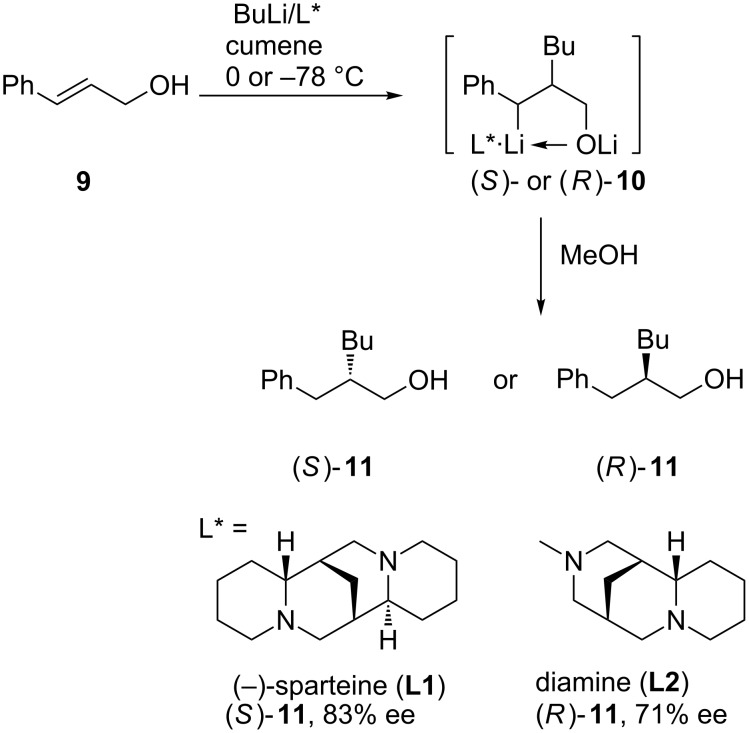
Carbolithiation of cinnamyl alcohol.

More recently, enantioselective carbolithiation of cinnamyl alcohol has been reinvestigated by using (+)-sparteine surrogates, such as diamine **L2** [12]. The best results are obtained by the treatment of cinnamyl alcohol (**9**) with the complex of butyllithium/diamine **L2** in cumene at 0 °C, obtaining alcohol (*R*)-**11** in 71% yield with 71% ee (Scheme 3). This is essentially opposite to the enantioselectivity obtained previously with (−)-sparteine **L1** (82% yield, 83% ee in favor of (*S*)-**11**) [10].

**Scheme 4:**
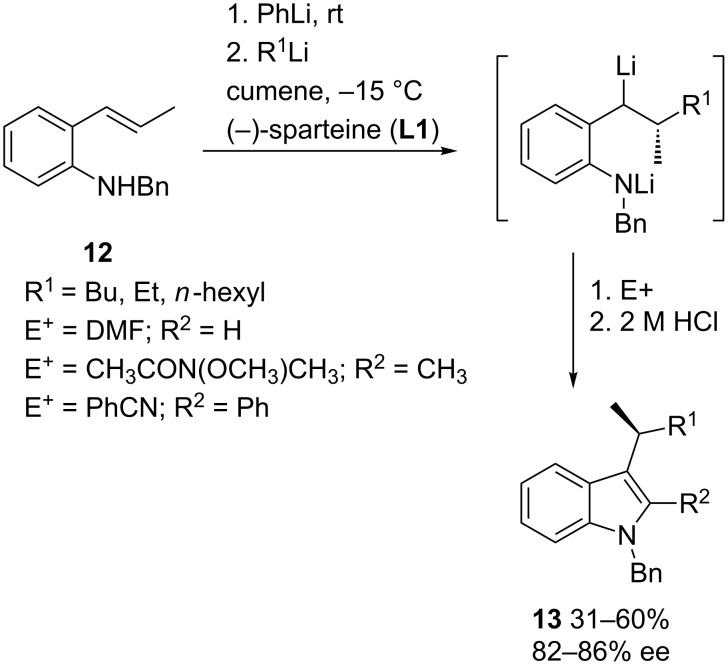
Carbolithiation of styrene derivatives.

Substituted styrene derivatives also undergo efficient enantioselective carbolithiation in the presence of chiral diamines, when there are electron-donating groups (e.g., methoxy and dialkylamino) at the *ortho*- or *para-*positions of the benzene ring to stabilize the intermediate benzyllithiums, thus deactivating the double bond towards organolithium addition and avoiding polymerization. Thus, the (−)-sparteine-mediated enantioselective intermolecular carbolithiation of (*E*)-*N*-benzyl-2-(prop-1-enyl)aniline (**12**) and subsequent trapping of the intermediate organolithium with a suitable electrophile, followed by an in situ ring closure and dehydration generates the substituted indoles **13** with high enantioselectivities (enantiomeric excess up to 86%) (Scheme 4). Different functional groups can be introduced at the C-2 position of the indoles by varying the electrophile [13–14]. The procedure has also been extended to the synthesis of chiral aromatics and heteroaromatics, such as isoquinolines or benzofurans, though lower yields and ee were obtained [15].

**Scheme 5:**
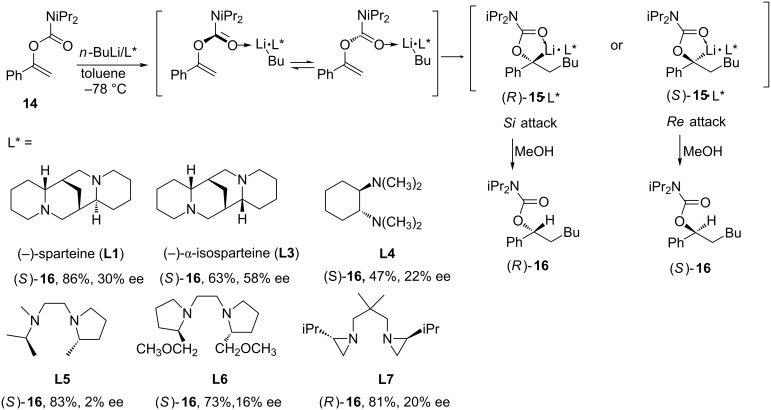
Carbolithiation of α-aryl *O*-alkenyl carbamates.

On the other hand, α-aryl *O*-(α-arylalkenyl) carbamates (α-carbamoyloxy-substituted styrenes) such as **14** undergo facile intermolecular carbolithiation reactions, via secondary α-carbamoyloxybenzyllithiums **15**, in the presence of chiral diamines, such as (−)-sparteine (**L1**) or (−)-α-isosparteine (**L3**), but only with moderate enantiofacial differentiation. The best results are obtained with unsubstituted alkenes (vinyl carbamates) by using butyllithium/(−)-α-isosparteine (up to 58% ee) (Scheme 5) [16]. The enantiofacial differentiation can be explained through the formation of the complexed conformers by the coordination of the organolithium and the chiral ligand, which will react with the double bond in an intramolecular *syn*-addition to form benzyllithium derivatives **15**. These secondary α-carbamoyloxybenzyllithiums are configurationally stable and can be trapped. The authors assume that the problem of these enantioselective carbolithiations is due to the interconversion of the conformers, formed by coordination of *n*-BuLi and the chiral ligand, being too slow, and that the energetic barriers are of the magnitude of the activation energies of the competing diastereomorphic addition steps.

**Scheme 6:**
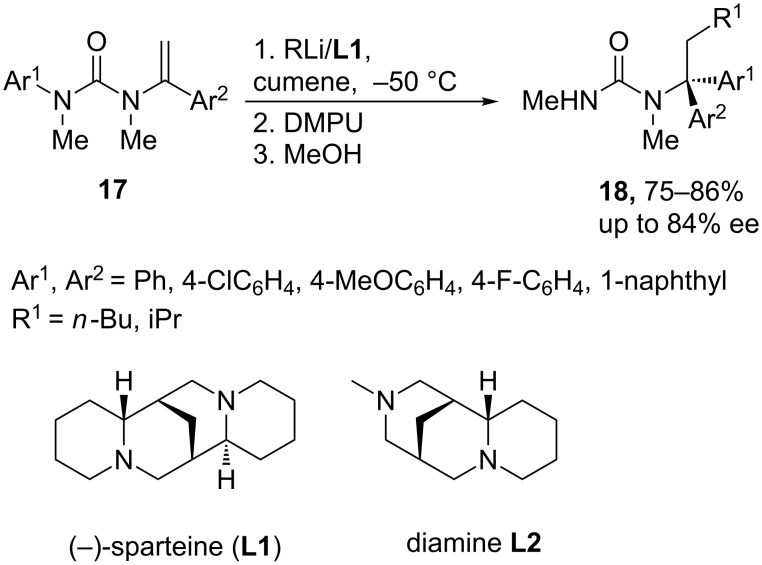
Carbolithiation-rearrangement of *N*-alkenyl-*N*-arylureas.

In the presence of (–)-sparteine (**L1**), *N*-alkenyl-*N*-arylureas **17** undergo addition of organolithiums to generate stabilized benzyllithiums, in which a N to C aryl transfer occurs by using DMPU as an additive to accelerate the aryl migration [17], probably by favoring the formation of solvated ion pairs [18]. Thus, this tandem enantioselective carbolithiation–rearrangement of vinylic ureas bearing an *N*-aryl group leads to the formation of two new carbon–carbon bonds with control of the absolute configuration, providing enantiomerically enriched amine derivatives **18** with a quaternary stereogenic center in the alpha position to the nitrogen atom (Scheme 6) [19]. The stereochemical outcome is explained taking into account that both protonation and aryl migration are stereochemically retentive, while carbolithiation is *syn* selective. The reactions begin with an asymmetric carbolithiation, in which the (−)-sparteine-complexed organolithium attacks the *Re* enantiotopic face of the alkene to form a stereodefined organolithium formed under kinetic control. On the other hand, the use of (+)-sparteine surrogate **L2** as a chiral ligand allows the synthesis of the products with the opposite absolute configuration, even when using THF as solvent [19].

**Scheme 7:**
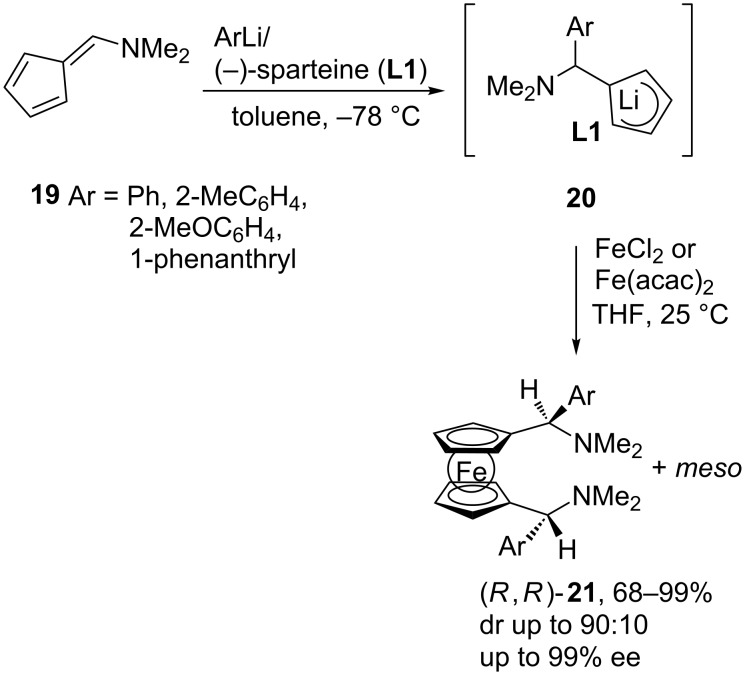
Carbolithiation of *N*,*N*-dimethylaminofulvene.

The carbolithiation reaction of 6-(*N*,*N*-dimethylamino)fulvene (**19**) with aryllithiums can be carried out in an enantioselective fashion in the presence of (−)-sparteine (**L1**). Thus, addition of the aryllithiums (generated from the corresponding aryl bromides and lithium metal) to **19** occurs cleanly at −78 °C in toluene, and the resulting chiral cyclopentadienyllithiums **20** are transformed into ferrocenes **21** by treatment with FeCl_2_ or Fe(acac)_2_ (Scheme 7). The enantiomeric excess of the ferrocenes is very high, up to 91% ee for those containing one chiral side chain and 99% ee or higher for those containing two chiral side chains, one on each of the cyclopentadienyl ring [20]. The chiral ferrocenes obtained are versatile synthetic intermediates that can be converted into various types of chiral ferrocene derivatives through diastereoselective *ortho*-lithiation [21].

**Scheme 8:**
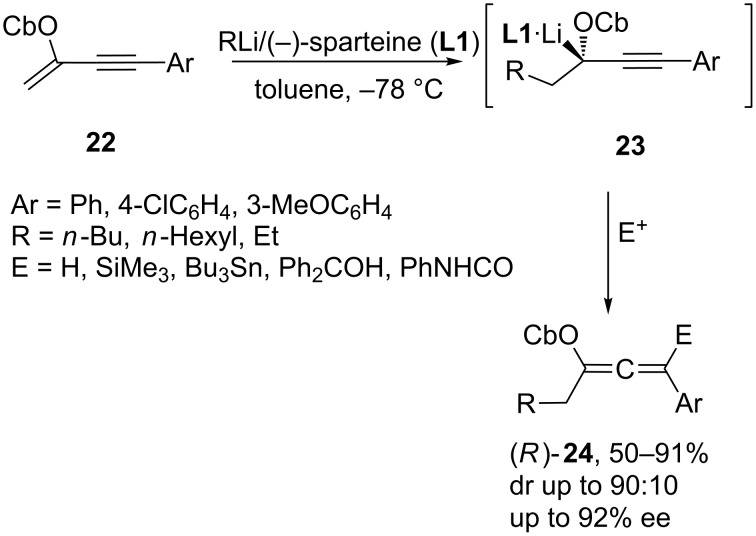
Carbolithiation of enynes.

Some years ago, the concept of flash chemistry was proposed, involving fast chemical synthesis by using flow microreactors, which enabled the use of highly reactive intermediates before they decompose [22–25]. Thus, Yoshida [26] demonstrated that the addition of aryllithiums, generated by halogen–lithium exchange, to conjugated enynes bearing an appropriate directing group occurs with complete regioselectivity and in good yields. More recently, they applied this concept to avoid the epimerization of reactive intermediates, which has allowed them to carry out the enantioselective version of the above procedure. Thus, the use of a flow microreactor system has allowed the enantioselective carbolithiation of conjugated enynes, followed by the reaction with electrophiles to give enantioenriched chiral allenes. By high-resolution control of the residence time, the epimerization of a configurationally unstable chiral organolithium intermediate **23** could be suppressed. Using this method, *n*-butyllithium reacts with enynes **22** in the presence of chiral ligands, and the resulting organolithiums can be trapped with different electrophiles to afford allenes **24** with complete regioselectivity and good yields. The best ee is obtained when there is a carbamoyloxy group (CbO) as directing group in the substrate with (−)-sparteine (**L1**) as the chiral ligand (Scheme 8) [27].

Despite the success achieved in the procedures described above, stoichiometric use of chiral ligands is usually required. However, recently a general catalytic methodology for the enantioselective intermolecular addition of alkyllithiums has been reported, though it implies transmetalation to copper complexes. In this case, the use of a copper-base chiral catalytic system allows carbon–carbon bond formation by allylic alkylation with alkyllithiums, with high enantioselectivities and high functional-group tolerance [28–29]. This process may open new interesting applications in organolithium chemistry, though it is beyond the scope of this review.

### Intramolecular carbolithiation reactions

**Scheme 9:**
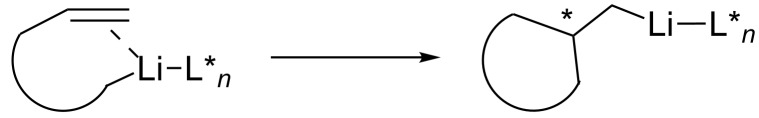
Intramolecular carbolithiation.

In the intramolecular carbolithiation of alkenes the highly reactive organolithium species has to be selectively generated in the presence of the internal alkene. Several approaches have been used for this purpose, such as halogen–lithium exchange, tin–lithium exchange, selenium–lithium exchange or reductive lithiation [1–8]. Once the organolithium has been generated, the intramolecular carbolithiation reaction usually takes place with high stereochemical control, as a consequence of a rigid transition state in which the lithium atom is coordinated to the remote π-bond [30–32]. This high stereocontrol has allowed the synthesis of enantiomerically pure carbocycles and heterocycles through diastereoselective cyclization of chiral nonracemic substrates [33–38]. Additionally, the internally coordinated organolithium would have two additional sites available for coordination with a chiral bidentate ligand. Thus, enantioselective versions of intramolecular carbolithiation reactions can also be carried out under the influence of a chiral ligand for lithium (Scheme 9).

**Scheme 10:**
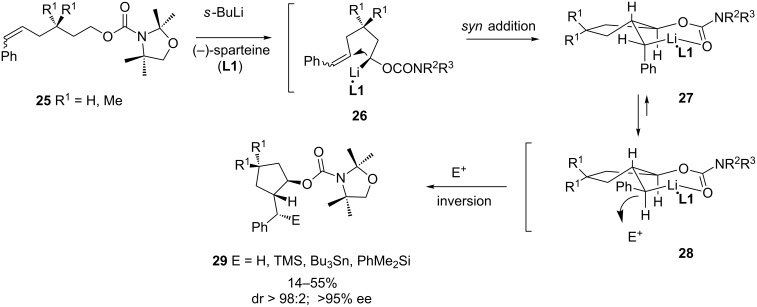
Carbolithiation of 5-alkenylcarbamates.

**Scheme 11:**
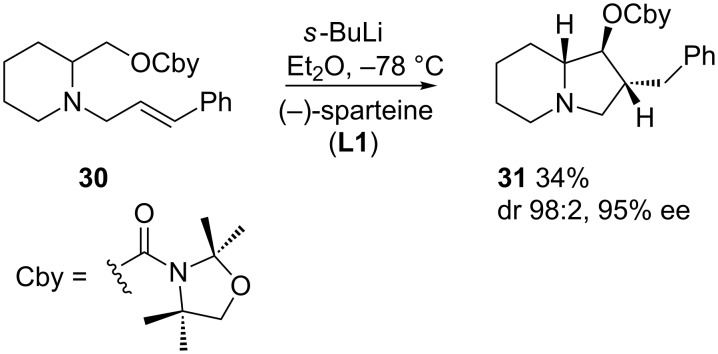
Carbolithiation of cinnamylpiperidines.

However, the first examples reported of asymmetric intramolecular carbolithiation reactions using alkyllithiums took advantage of the (−)-sparteine-mediated enantioselective deprotonation reaction of carbamates [39]. Thus, achiral (*Z*)- or (*E*)-6-phenyl-hex-5-enylcarbamates **25** can be cyclized with *sec-*butyllithium in the presence of (−)-sparteine (**L1**) at −78 °C giving *trans*-substituted cyclopentanes **29** in high diastereo- and enantioselectivity. The primary benzyllithium intermediates are also diastereoselectively substituted by different electrophiles creating a third consecutive stereogenic center (Scheme 10) [40]. The stereochemical outcome is explained assuming that the (−)-sparteine-mediated enantioselective deprotonation leads to an (*S*)-configurated (α-carbamoyloxy)alkyllithium intermediate **26**. Then, the cycloisomerization occurs through a *syn* addition to the π-bond to give a stabilized benzyllithium **27**, which epimerizes to a more stable benzyllithium **28**, due to the equatorial position of the phenyl substituent, which is subsequently trapped by electrophiles with inversion. This proposal is supported by the fact that both *E*- and *Z*-alkenes led to the same diastereomers. This procedure has been further extended to 4-functionalized 6-phenyl-hex-5-enylcarbamates [41]. Following a similar approach, deprotonation of racemic (carbamoyloxy)methyl-*N*-cinnamylpiperidine **30** with *s*-butyllithium/(−)-sparteine (**L1**), and subsequent anionic 5-*exo-trig* cyclization, leads to indolizidine **31** with high diastereomeric and enantiomeric ratios, in moderate yield. (Scheme 11) [42]. The resulting benzyllithium can also be trapped with electrophiles, though the stereoselectivity in this fourth center is not so high, and depends largely on the electrophile.

**Scheme 12:**
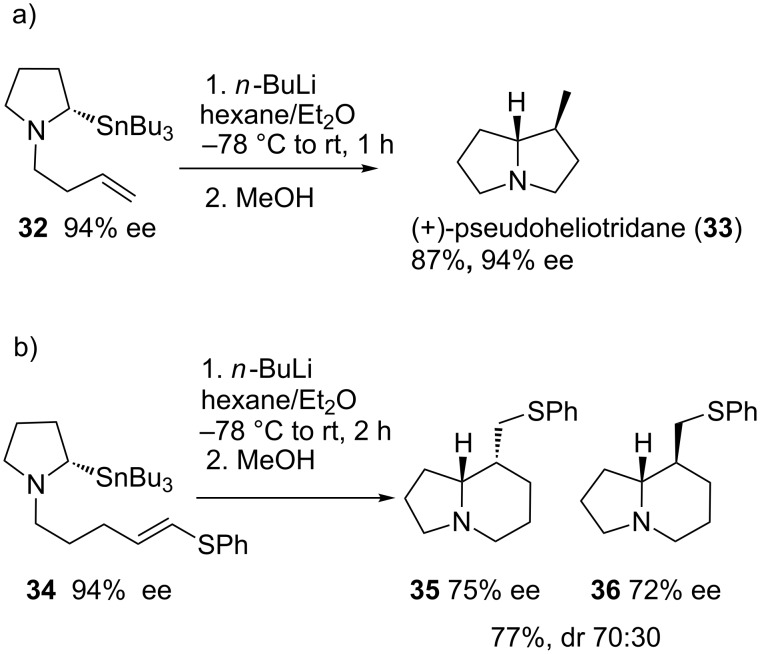
Carbolithiation of alkenylpyrrolidines.

However, in these examples, the chiral ligand induces the enantioselective deprotonation to generate a nonracemic organolithium, which adds diastereoselectively to the carbon–carbon double bond. In this context, configurationally stable secondary α-amino alkyllithiums have also been obtained by tin–lithium exchange (Scheme 12). Addition to the double bond with complete retention of configuration affords a new organolithium, which can be reacted with electrophiles to afford pyrrolizidines with no loss of optical purity. Scheme 12a shows the application to the synthesis of the pyrrolizidine alkaloid (+)-pseudoheliotridane (**33**) [43]. The reaction can be extended to the formation of six-membered rings, though the slow cyclization rate results in racemization. The use of a phenylthio-substituted alkene favors the cyclization, and the faster reaction rate results in an improved optical purity of the indolizidines **35** and **36**, though with moderate diastereo- and enantioselectivity. The addition of TMEDA increases the rate of racemization, resulting in an inversion of diastereoselectivity to obtain **36**, albeit in racemic form [44].

**Scheme 13:**
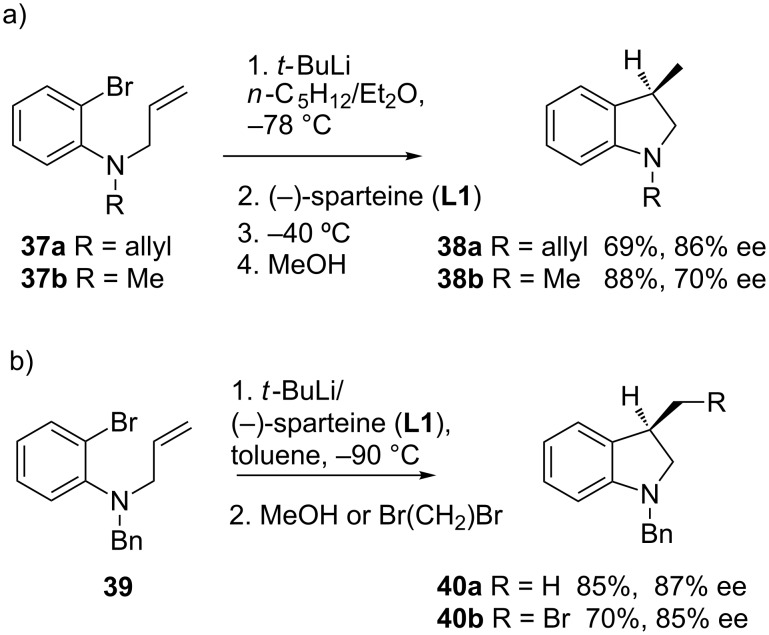
Enantioselective carbolithiation of *N*-allyl-2-bromoanilines.

The enantioselective cycloisomerization of achiral organolithiums in the presence of a chiral ligand has been reported with aryllithium reagents, which can be efficiently prepared by halogen–lithium exchange. Hence, the enantioselective intramolecular carbolithiation of *N*-allyl-2-bromoanilines by using *tert*-butyllithium in the presence of (−)-sparteine (**L1**) for the synthesis of 3-substituted indolines, was reported simultaneously by Bailey [45] and Groth [46]. Thus, (*R*)-(−)-1-allyl-3-methylindoline (**38a**) was obtained in high yield and ee by reaction of *N*,*N*-diallyl-2-bromoaniline (**37a**) with *t-*BuLi in *n*-pentane/diethyl ether in the presence of (−)-sparteine (**L1**) (Scheme 13a). The choice of solvent has an important effect on the enantioselectivity, favoring the coordination of the ligand with lithium. While solvent systems composed of hydrocarbon/diethyl ether are effective, the use of THF resulted in almost complete loss of enantioselectivity. The cyclization of amine **37b** under identical conditions was less enantioselective than the analogous diallyl substrate **37a** (70% versus 86%) [45]. On the other hand, *N*-allyl-*N*-benzyl-2-bromoaniline (**39**) also undergoes enantioselective intramolecular carbolithiation with *t*-BuLi/(−)-sparteine (**L1**) in toluene as solvent (Scheme 13b) [46].

**Scheme 14:**
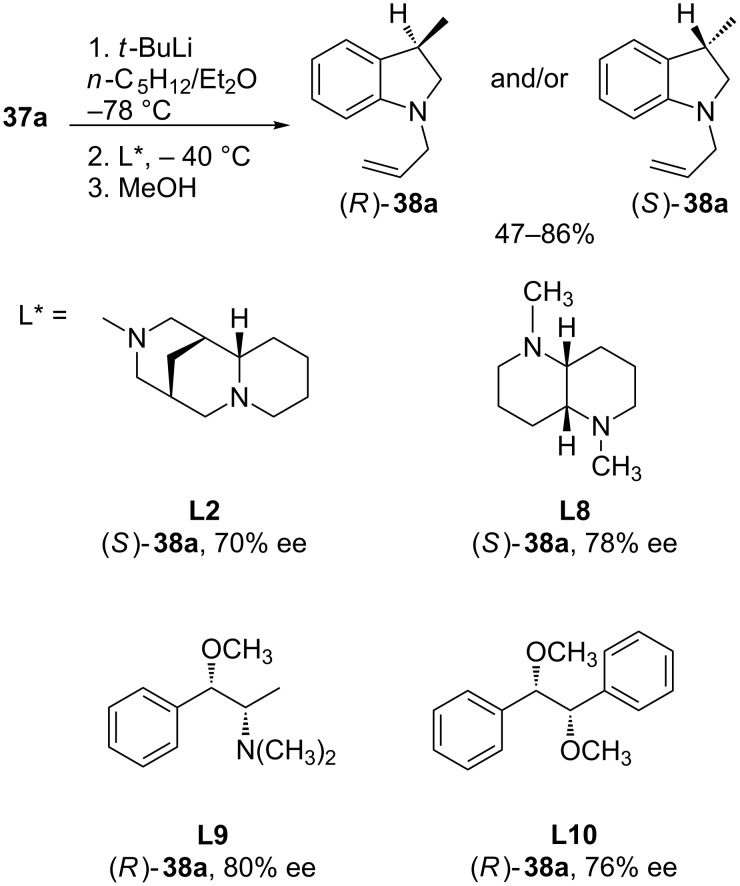
Effect of the ligand in the carbolithiation reaction.

The ability of a large and chemically diverse set of thirty chiral ligands to effect the asymmetric intramolecular carbolithiation of *N*,*N*-diallyl-2-bromoaniline (**37a**) has been investigated in an attempt to elucidate the structural motifs required to provide high enantiofacial selectivity in the ring closure [47]. Although none of the ligands examined affords 1-allyl-3-methylindoline (**38a**) in significantly higher enantiomeric excess than previously observed with (−)-sparteine (**L1**), several ligands available in either enantiomeric form match its utility in this chemistry (Scheme 14). Among ethers and aminoethers, ligands (1*S*,2*S*)-*N*,*O*-dimethylpseudoephedrine (**L9**) and (1*S*,2*S*)-1,2-dimethoxy-1,2-diphenylethane (**L10**) are particularly effective surrogates for sparteine, affording 3-methylindoline (*R*)-**38a** in good yield and high enantiomeric excess (Scheme 14). With regard to chiral diamine ligands, enantiomeric excess is only maintained by using *cis*-1,5-diazadecalin **L8**, leading to the indoline of opposite configuration. Unfortunately, this diamine is not commercially available, and its synthesis requires resolution. More recently, O’Brien [48] has reported that (+)-sparteine surrogate **L2** gave a similar degree of enantioselectivity and indoline (*S*)-**38a** of 85:15 er (84% yield) was produced (Scheme 14).

**Scheme 15:**
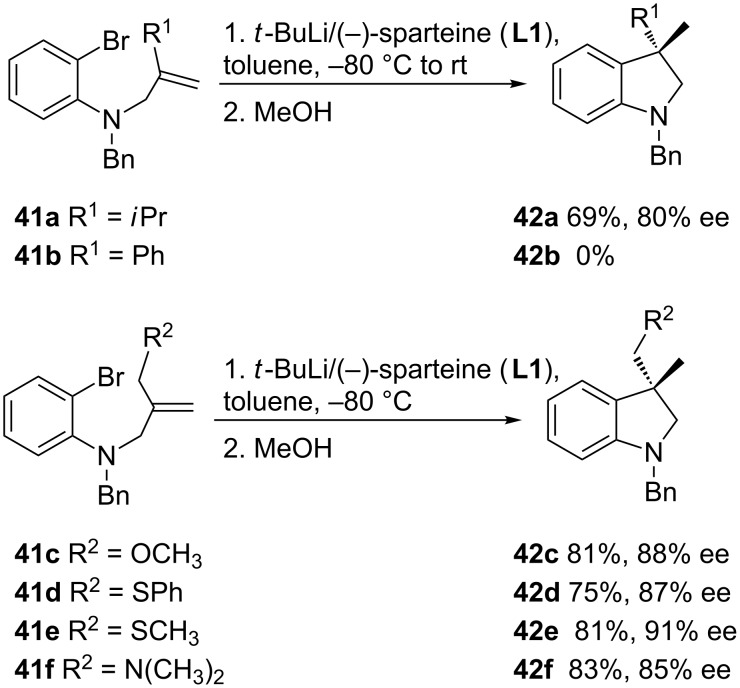
Effect of the alkene substitution in the carbolithiation reaction.

Substituted alkenes may also be used in these reactions, and 3,3-disubstituted indolines **42** are obtained in moderate to high enantiomeric excesses, depending on the nature of the substituent on the alkene (Scheme 15) [49]. The steric demand of an isopropyl group increases the enantioselectivity in the cyclization of **42a** (Scheme 15), though the reaction fails with a phenyl substituent (**42b**). A chelating donor substituent in the allylic moiety may assist the carbolithiation reaction. The best results regarding both chemical yields and enantioselectivity are obtained when R^1^ = OMe, SPh, SMe, and NMe_2_, at −80 °C in toluene as solvent.

**Scheme 16:**
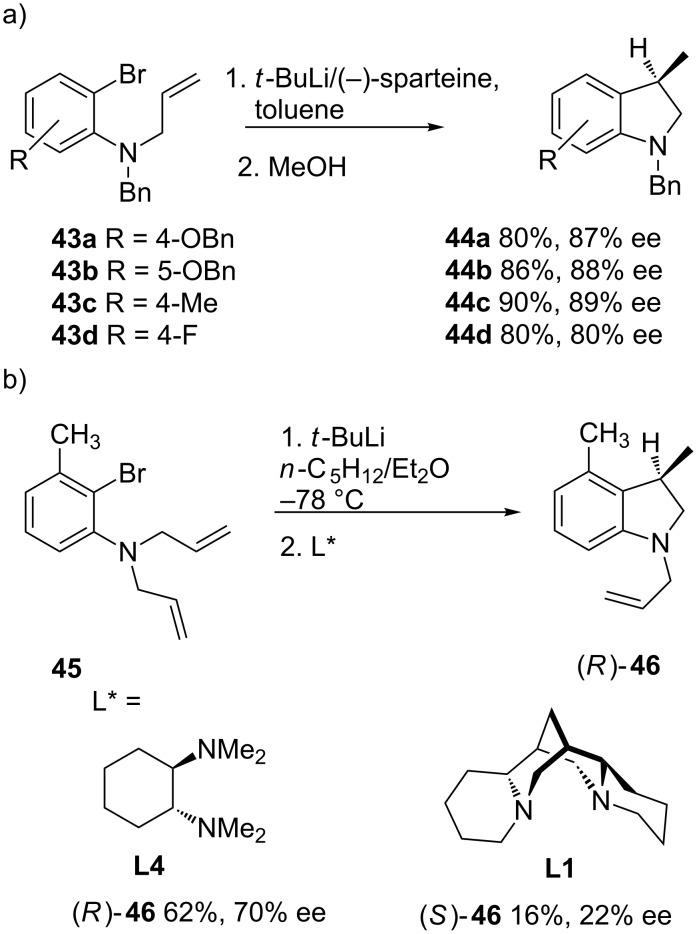
Effect of the ring substitution in the carbolithiation reaction.

The influence of the substitution pattern in the aromatic ring on both the yield and the enantioselectivity is not clearly established. The introduction of electron-deficient substituents may require higher temperatures for the reaction to proceed to completion but maintain good enantioselection (Scheme 16a) [46]. On the other hand, a seemingly minor variation in substrate structure may have a pronounced effect on the ability of a given ligand to facilitate cyclization. Thus, the presence of a substituent in the position *ortho* to the lithium atom leads to lower yields and the opposite enantiomer (*S*)-3-methylindoline (*S*)-**46** with low enantiomeric excess (22% ee) when (−)-sparteine (**L1**) is used (Scheme 16b). However, in this case, the (1*R*,2*R*)-*N*,*N*,*N*’,*N*’-tetramethylcyclohexane-1,2-diamine (**L4**) proves to be a more efficient ligand for lithium, leading to (*R*)-3-methylindoline (*R*)-**46** in 70% ee [47].

**Scheme 17:**
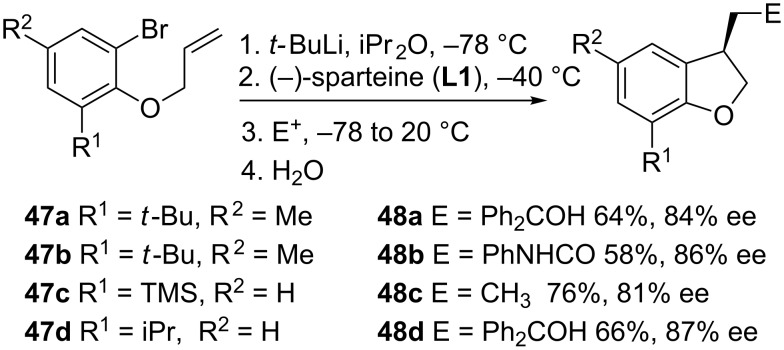
Enantioselective carbolithiation of allyl aryl ethers.

This type of intramolecular carbolithiation is also useful for the synthesis of fused furan systems. Thus, enantiomerically enriched 2,3-dihydrobenzofurans **48** are obtained in moderate to good yields and high enantiomeric purity by using (−)-sparteine (**L1**) as chiral ligand, and diisopropyl ether as solvent (Scheme 17). The resulting organolithium can be trapped with several external electrophiles [50]. The presence of a substituent at the 6-position of the aromatic ring (R^1^ ≠ H) is necessary to obtain the benzofurans **48**. Otherwise, the aryllithium intermediate undergoes a tandem carbolithiation–γ-elimination leading to enantioenriched 2-cyclopropylphenols [51].

**Scheme 18:**
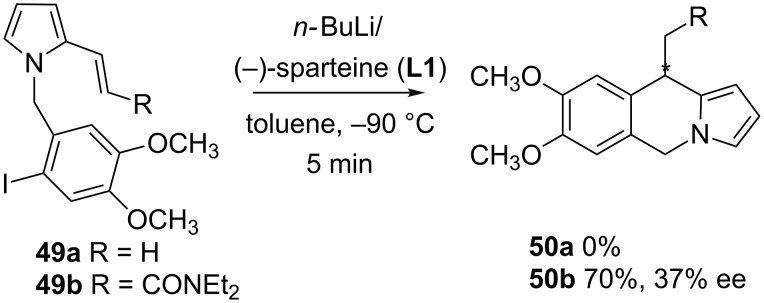
Formation of six-membered rings: pyrroloisoquinolines.

As shown, these enantioselective intramolecular cyclizations are mostly employed for the formation of five-membered rings. The 6-*exo* cyclization of an aryllithium intermediate is also possible, but generally the alkene has to be substituted with a stabilizing group for the resulting organolithium to favor the cyclization, as has been shown before for alkyllithium derivatives (Scheme 12b) [44]. This type of reaction has been used, for instance, for the diastereoselective synthesis of enantiomerically pure isoquinoline rings [34,37], but just a few examples of the enantioselective variant have been reported so far. Thus, as shown on Scheme 18, the cyclization of the aryllithium generated from **49a** does not occur on the unsubstituted alkene, whereas the introduction of an amide group favors a fast cyclization at low temperature, and pyrroloisoquinoline **50b** is isolated in good yield. However, this higher reactivity results in a low enantioselection when (−)-sparteine (**L1**) is used as chiral ligand, under various reaction conditions [34].

**Scheme 19:**
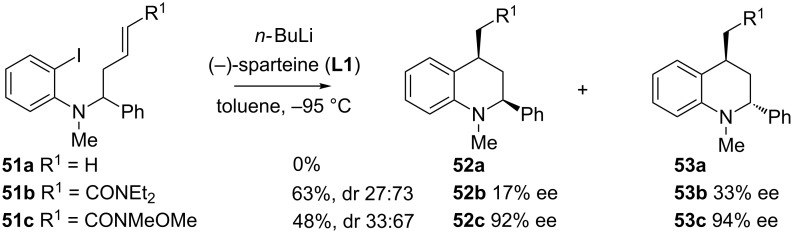
Formation of six-membered rings: tetrahydroquinolines.

Similarly, the intramolecular carbolithiation of *N*-alkenyl substituted *o*-iodoanilines **51** affords enantiomerically enriched 2,4-disubstituted tetrahydroquinolines **52** and **53** by using (−)-sparteine (**L1**) as chiral ligand [52]. An amide group is also required to favor the cyclization, and also has an important effect in both the diastereoselectivity and the enantioselectivity. As shown on Scheme 19, the best results in terms of enantioselectivity were obtained by using Weinreb amide **51c**, though with moderate diastereoselectivity.

## Conclusion

As has been shown through these examples, the enantioselective carbolithiation reaction is an attractive approach for the construction of carbon–carbon bonds. However, the applicability of this method is not yet general. Among the different types of ligands used, chiral bidentate diamines, and especially (−)-sparteine, are the most generally employed. However, the structural requirements of the chiral ligand required to provide high enantiofacial selectivity for a broader range of substrates are still unclear. The intermolecular reactions usually take advantage of chelation and stabilizing effects on the resulting organolithium to obtain high enantioselectivity and avoid polymerization. Enantiomerically enriched five-membered rings can be prepared through intramolecular carbolithiation reactions of unsaturated aryllithiums in the presence of chiral ligands. The application to the formation of six-membered or larger cycles with the same degree of stereo- and regiochemical efficiency has not been fully developed. On the other hand, the high reactivity of the organolithiums requires the use of stoichiometric amounts of the chiral ligand.
